# Room-Temperature Electron Spin Generation by Femtosecond Laser Pulses in Colloidal CdS Quantum Dots

**DOI:** 10.3390/ma6104523

**Published:** 2013-10-15

**Authors:** Haifang Tong, Donghai Feng, Xiao Li, Li Deng, Yuxin Leng, Tianqing Jia, Zhenrong Sun

**Affiliations:** 1 State Key Laboratory of Precision Spectroscopy, East China Normal University, Shanghai 200062, China; E-Mails: tonghaifang2056@163.com (H.T.); lixiao_1003@126.com (X.L.); ldeng@phy.ecnu.edu.cn (L.D.); tqjia@phy.ecnu.edu.cn (T.J.); zrsun@phy.ecnu.edu.cn (Z.S.); 2 State Key Laboratory of High Field Laser Physics, Shanghai Institute of Optics and Fine Mechanics, Chinese Academy of Sciences, Shanghai 201800, China; E-Mail: lengyuxin@mail.siom.ac.cn

**Keywords:** electron spin, optical manipulation, pump-probe, quantum dots

## Abstract

We present an experimental investigation of optical spin orientation in colloidal CdS quantum dots (QDs) by a femtosecond laser pulse at room temperature. The spin carrier and its spin-generation process are clarified. Firstly, the observed spin signals of CdS QDs in time-resolved Faraday rotation measurements are shown to belong to electron carriers, by comparing the spin dephasing dynamics and Landé g factor between CdS QDs and bulk materials. Secondly, spin dynamics unaffected by the faster carrier recombination suggests that the spin-polarized electrons are not photoexcited but resident in the dots. Moreover, hole spins should dephase very fast compared with electron spins, otherwise the trion (two electrons with opposite spin orientations and one hole) recombination process will affect the resident electron spin signals. The electron spin is generated in a short time of which the excitation light is absorbed and the resident electron is excited to trion states, *i.e.*, of pulse durations. Due to fast hole spin dephasing, trion recombination gives null spin signals, and the subsequent electron spin dynamics is controlled by its intrinsic mechanisms.

## 1. Introduction

The spin states of semiconductor quantum dots carry prospects in realization of solid-state qubits for quantum-information processing and computation (QIPC) [1,2]. A key element for QIPC is the initial quantum state preparation. As quantum error correction (QEC) schemes require >10^4^ operations within the decoherence time, the state initialization speed must be much faster than the quantum state decoherence rate [3]. In this regard, ultrashort laser pulses have advantages in fast spin generation and control, as well as convenient detection of transient spin states [3,4,5,6,7,8,9]. Applications of ultrafast laser technology in semiconductor spintronics have attracted much interest in recent years.

III-V semiconductor (such as InAs, GaAs, InGaAs) quantum dots (QDs), often used in spin investigations, normally have an ensemble spin dephasing time $T_{2}^{*}$ of a few nanoseconds at cryogenic temperature [7]. Spin dephasing time in QDs is limited by electron-nuclear hyperfine interactions in zero or low magnetic field [10,11]. Thus, materials with weak hyperfine coupling are helpful to hold a long electron spin lifetime. II-VI QDs generally have smaller hyperfine-interaction strength than that of their III-V counterparts, and consequently draw much attention in spin manipulation studies [12,13,14,15]. Recently, colloidal II-VI CdS QD has been shown to keep long-lived spin coherence with $T_{2}^{*}>3\text{ ns}$ at room temperature [16]. Room-temperature environment in spin manipulations would be of great interest for future practical device applications. The spin coherence amplitude in colloidal CdS QDs could be easily manipulated by a prepump or control laser pulse with femtosecond pulse durations [17], which further makes CdS QD materials attractive in spin studies.

In this work, we present an in-depth study to clarify the spin generation process in colloidal CdS QD at room temperature, under the irradiation of femtosecond laser pulses. We will firstly clarify whether the measured spin signals come from electrons or holes, and whether the spin carriers are photoexcited or resident in the dots. The answer is that the spin signal comes from resident electrons. Then, we experimentally judge that the spin relaxation time of the hole in trion complexes (two electrons and one hole) should be much faster than the trion recombination time. Based on these experimental results, the spin generation process will finally be illustrated in detail.

## 2. Experimental

**Figure 1:**
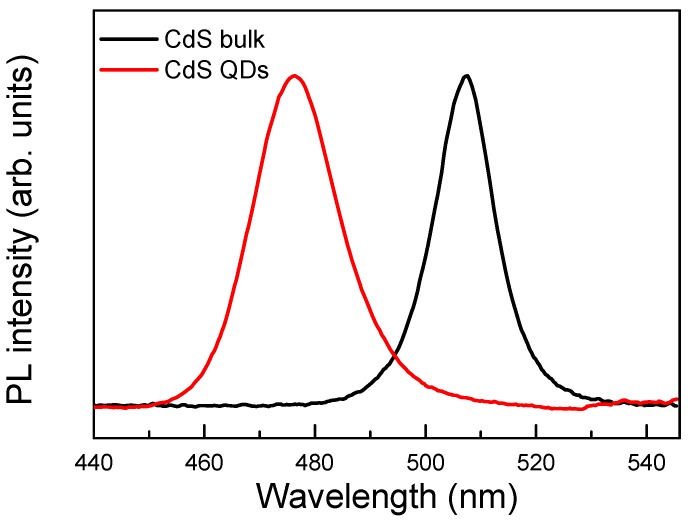
Photoluminescence spectra for CdS quantum dots (QDs) and bulk samples. Peak intensities are normalized to the same level.

Commercially available colloidal CdS QDs were purchased from Sigma-Aldrich Corporation (St. Louis, MO, USA). The used QD sample (Lumidot™ CdS 480) is surface-stabilized with oleic acid coating, and dissolved in toluene with dot diameter sizes ~5.6 nm. The QD’s photoluminescence (PL) peak is at ~476 nm. The obtained QDs solution is used in a quartz cell directly for the measurements. As a comparison, we also measured the electron spin coherence in bulk CdS material [single crystals with a thickness of 0.5 mm, purchased from MTI Corporation (Richmond, CA, USA)]. The PL peak of CdS single crystals is at ~507 nm. The PL spectra for CdS QD and bulk samples are shown in Figure 1. Due to quantum confinement effect, the QD emission peak is blue shifted compared with that of bulk sample.

**Figure 2:**
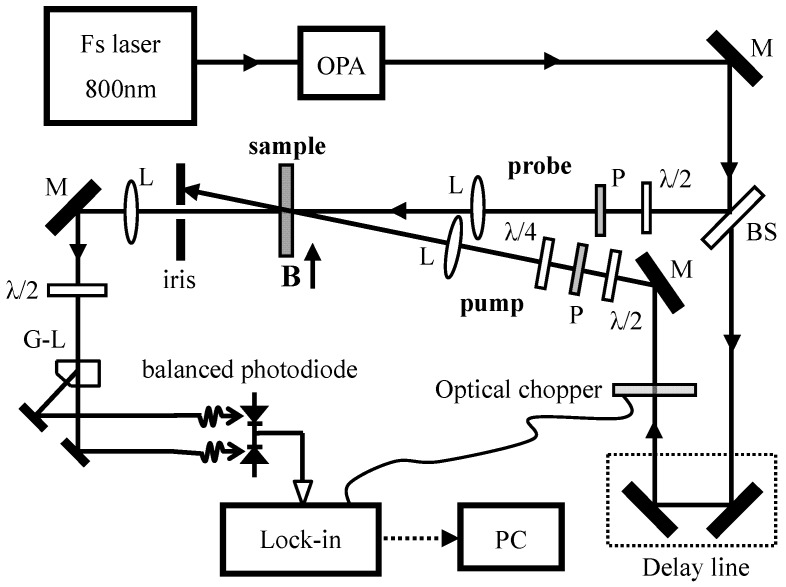
Experimental scheme for time-resolved Faraday rotation measurements.

The main measurement techniques involved in this paper are time-resolved Faraday rotation (TRFR) and time-resolved differential transmission (TRDT) spectroscopies, which are used to measure spin and carrier relaxation dynamics, respectively. Figure 2 shows the experimental setup for TRFR measurements. Femtosecond visible laser pulses come from an optical parametric amplifier (OPA), obtained by parametric nonlinear processes and frequency mixing from a Ti: sapphire regenerative amplifier (800 nm wavelength, 50 fs pulse duration, 3 mJ/pulse intensity, and 1 kHz repetition rate). The OPA output is split by a beam splitter into pump and probe parts. The energies of the pump/probe laser beams are degenerate, which are set in the lower energy side to the PL peak for both CdS QD and bulk samples. The circularly-polarized pump and linearly-polarized probe laser beams are focused into a same spot with a diameter of about 200 μm on the sample. Their intensity can be varied by adjusting a half-wave plate before a Glan-Laser polarizer. The probe intensity is 10 times lower than the pump intensity.

Circularly polarized pump laser pulse generates spin-polarized carriers, and the generated spin signal is detected by the rotation angle of the polarization plane of a linearly polarized probe pulse. Magnetic field *B* up to 1 tesla from an electromagnet is applied in Voigt geometry (*B* perpendicular to spin polarization direction). The measurements are done using a balanced photodiode detector connected to a lock-in amplifier in order to get a better signal to noise ratio. In the TRFR measurements, the broadband half-wave plate rotates the transmitted probe beam polarization to 45° with respect to the Glan-Laser polarizing beam splitter before pumping-on, getting a null signal in the difference of bridge arms. The small pump-induced deviations from this null condition then represent the polarization plane rotation of the probe light. The detection scheme using a balanced diode bridge helps to cancel out all laser intensity noises effectively. TRDT setup is similar to that for TRFR. The mere difference is that the transmitted probe light does not go through the Glan-Laser polarizing beam splitter, but, instead, goes directly into a photodiode for lock-in detection. All the measurements are performed at room temperature.

## 3. Results and Discussion

**Figure 3:**
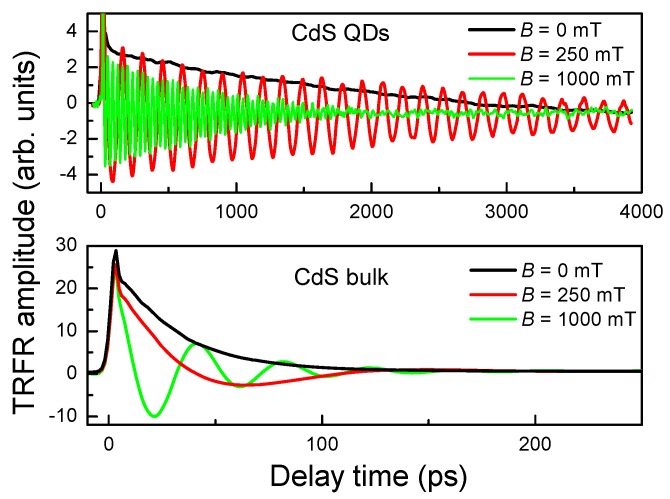
Time-resolved Faraday rotation signals for CdS QD and bulk sample in different transverse magnetic fields.

Figure 3 shows the TRFR measurement results both in CdS QDs and in bulk samples at *B* = 0, 250 and 1000 mT. Compared with that in a zero magnetic field, the signals show periodic oscillation in an external transverse magnetic field, denoting the existence of spin signals. The signals oscillate as a result of spin precession in the magnetic field, with a frequency being equal to Larmor precession frequency, $\omega_{L}=g\mu_{B}B/\hbar$. The oscillatory amplitude decays with time due to spin dephasing by interaction with the environment. The evolution of Faraday rotations θ*_F_*(*t*) can be described by Equation (1),

(1) $$\theta_{F}(t)=\theta_{F}(0)\cos(\omega_{L}t)\exp(-\frac{t}{T_{2}^{*}})$$

where θ*_F_*(0) is the initial amplitude at the time of which spins are just initiated by the pump pulse, and $T_{2}^{*}$ is the ensemble spin dephasing time.

**Figure 4:**
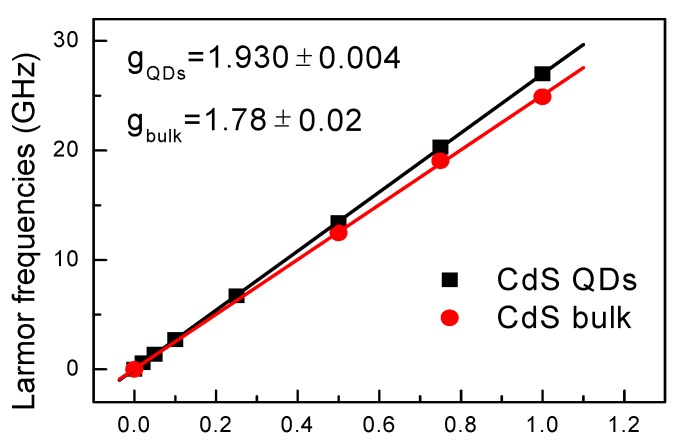
Magnetic field dependence of time-resolved Faraday rotation (TRFR) oscillation frequencies. Linear fits give information of g factor, with error obtained from the fit algorithm.

With increasing magnetic field, the Faraday rotation (FR) oscillatory frequency becomes larger. Figure 4 gives information of Larmor frequency as a function of magnetic field for CdS QDs and bulk sample. According to $g=\hbar \omega_{L}/(\mu_{B}B)$, *g* = 1.930 ± 0.004 and 1.78 ± 0.02 can be obtained for QDs and bulk sample, respectively. The g factor in CdS bulk sample agrees well with the value of electron g factor in the literature (while hole g factor is around 1.23 in CdS bulk samples) [18], denoting the measured spin signals are from electron carriers. The *g* value of 1.93 in CdS QDs is also electronic, and larger than 1.78 in CdS bulk due to that quantum confinement in QDs makes electron g factor become larger [19].

In CdS QDs, $T_{2}^{*}$ are varied remarkably in different magnetic fields as shown in Figure 3, from 0.7 ns at *B*= 1000 mT to 2.8 ns at *B*= 250 mT. However, $T_{2}^{*}$ of CdS QDs are all obviously longer than that of CdS bulk sample which is approximately 36 ps in all measured magnetic fields. The reason is that spin-orbit coupling mechanism, which controls the spin dynamics in bulk materials, is strongly suppressed in QDs due to electronic localization on the nanometer length scale.

**Figure 5:**
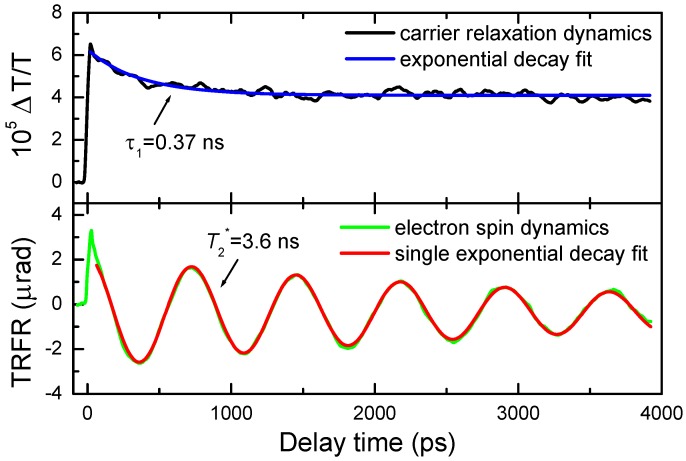
Comparison between time-resolved differential transmission and Faraday rotation measurement results under identical experimental conditions.

The electron could be photo-excited or originally resident in the QD. The spin lifetime of photo-excited electrons will be limited by the electron-hole recombination process, but not in the case of resident electrons. Therefore, we can compare the decay dynamics between TRFR and TRDT measurements in order to check the origin of spin carriers, where spin and carrier relaxation dynamics are detected, respectively. Figure 5 shows the results of TRDT and TRFR measurements at *B*= 50 mT, under the same pump and probe light conditions. In the TDRT measurements, the curve shows biphasic dynamics, with τ_1_ ~ 0.37 ns and an immeasurably long time τ_2_ (τ_1_ is related to the intrinsic recombination of initially populated internal core states and *τ*_2_ related to the recombination of surface trapped states [20]), while the TRFR curve only shows single exponential dynamics with $T_{2}^{*}\text{\textasciitilde{}}3.6\text{ ns}$. The carrier population dynamics has no effect on the spin evolution process, otherwise the spin dynamics would be partly affected by *τ*_1_ process of the carrier recombination. Thus, the spin carriers in our QD samples should be resident electrons. The resident electron may come from the charge transfer between the stabilizer reagent of oleic acid molecules and CdS QDs [20].

**Figure 6:**
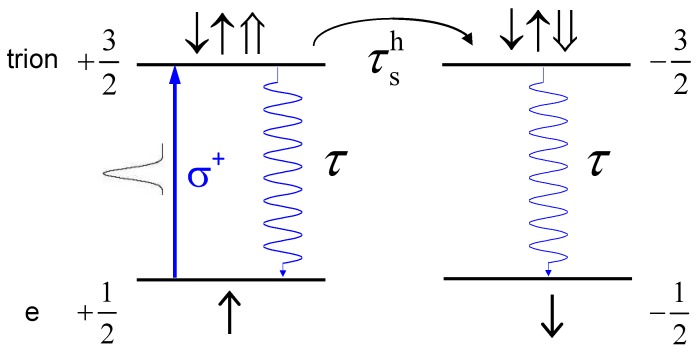
Excitation scheme of the electron spin generation in a negatively charged quantum dot under resonant trion excitation. ↑↓ electron spin; ⇑,⇓, hole spin. $\tau ,{\;\tau }_{s}^{h}$, trion recombination time and hole spin lifetime, respectively.

Now we can figure out the excitation profile of a QD with a resident electron, under the irradiation of a circularly-polarized pump pulse. Optical excitation of a resident electron results in the formation of trions. The trion singlet ground state consists of two electrons with opposite spin orientations and a single hole with a spin of ±3/2. Let us consider the simplest case as shown in Figure 6, *i.e.*, the resonant excitation of the trion singlet ground state, by a σ^+^ laser pulse in zero magnetic field. According to optical selection rules, only spin-up resident electrons will be excited to spin-up trion states where the total spin is defined by the hole spin, leaving a net part of spin-down-polarized resident electrons [21]. When an external transverse magnetic field is applied, the spin-polarized electrons then precess, inducing periodically oscillatory FR signals.

The resultant spin-up trions can recombine to create spin-up electrons via the same channel, or spin-down electrons after the hole spin flip. If a hole in the trion looses its spin rapidly, trions will not contribute any Faraday rotation signals, and the spin polarization of the electrons returning from the trion recombination is also negligible. In this case, trion recombinations will not affect the resident electron spin dynamics. While if the hole spin relaxes slowly, e.g., much slower than carrier recombination rate, the resident electron spin dephasing rate will become $\left(1/T_{2}^{*}+1/\tau_{1}\right)$, or it is implied that trion recombinations have influence on the resient electron spin dynamics. Following the comparison result shown in Figure 5, one can conclude that hole spins of CdS QDs really have a rapid relaxation rate (much larger than trion recombination rate), otherwise the electrons unequally return from the two arms of trion recombinations, and then affect the resident electron spin relaxation dynamics. It is further convinced by the fact that no trion (or hole) spin signals are detected on the present measurement time scale. The hole-spin relaxation is rapid due to valence band mixing [22].

## 4. Conclusions

Spin initialization process under the irradiation of a femtosecond laser pulse is revealed experimentally in colloidal CdS QDs at room temperature. By comparing the spin dephasing dynamics and Landé g factor between CdS QDs and bulk materials, it is concluded that the observed spin signals of CdS QDs belong to electron carriers. Comparison between time-resolved differential transmission and Faraday rotation measurements shows that carrier recombination has no effect on electron spin relaxation dynamics. Therefore, one can conclude that, on the one side, the electron is resident in the dots, and on the other side, hole spins relax rapidly. The resident electron spin dynamics is not affected by carrier recombinations but controlled by its intrinsic mechanisms, e.g., electron-nuclear hyperfine interaction or inhomogenous dephasing [16]. The electron spin initialization process is completed within the pulse durations. As the ensemble dephasing time in colloidal CdS QDs is of a few nanoseconds [16], the spin initialization time is much faster than its relaxation time.
